# Repositioning Drugs for Rare Diseases Based on Biological Features and Computational Approaches

**DOI:** 10.3390/healthcare10091784

**Published:** 2022-09-16

**Authors:** Belén Otero-Carrasco, Lucía Prieto Santamaría, Esther Ugarte Carro, Juan Pedro Caraça-Valente Hernández, Alejandro Rodríguez-González

**Affiliations:** 1 Centro de Tecnología Biomédica, Universidad Politécnica de Madrid, Boadilla del Monte, 28660 Madrid, Spain; 2 ETS Ingenieros Informáticos, Universidad Politécnica de Madrid, Boadilla del Monte, 28660 Madrid, Spain

**Keywords:** rare diseases, biomedical informatics, drug repositioning, DISNET knowledge base

## Abstract

Rare diseases are a group of uncommon diseases in the world population. To date, about 7000 rare diseases have been documented. However, most of them do not have a known treatment. As a result of the relatively low demand for their treatments caused by their scarce prevalence, the pharmaceutical industry has not sufficiently encouraged the research to develop drugs to treat them. This work aims to analyse potential drug-repositioning strategies for this kind of disease. Drug repositioning seeks to find new uses for existing drugs. In this context, it seeks to discover if rare diseases could be treated with medicines previously indicated to heal other diseases. Our approaches tackle the problem by employing computational methods that calculate similarities between rare and non-rare diseases, considering biological features such as genes, proteins, and symptoms. Drug candidates for repositioning will be checked against clinical trials found in the scientific literature. In this study, 13 different rare diseases have been selected for which potential drugs could be repositioned. By verifying these drugs in the scientific literature, successful cases were found for 75% of the rare diseases studied. The genetic associations and phenotypical features of the rare diseases were examined. In addition, the verified drugs were classified according to the anatomical therapeutic chemical (ATC) code to highlight the types with a higher predisposition to be repositioned. These promising results open the door for further research in this field of study.

## 1. Introduction

Rare diseases (RD) are pathologies that have a limited prevalence in the population. For a disease to be considered rare, in the case of Europe, it must affect fewer than 5 per 10,000 inhabitants. In the United States, a disease is considered rare when it affects fewer than 200,000 people [1]. There are many people affected by rare diseases around the world; more than 55 million people suffer from a rare disease only in Europe and the USA [2]. Most rare diseases have a chronic and frequently disabling nature. They involve a heterogeneous multisystem complexity in diagnostics and treatment, creating a unique challenge to our public health [3].

The total number of rare diseases is difficult to specify. According to the Orphanet (https://www.orpha.net (accessed on 3 February 2021)) database, one of the most powerful resources about rare diseases, around 7000 diseases are catalogued. Moreover, EURORDIS (https://www.eurordis.org (accessed on 15 April 2021)) statistics show that every year, around 250 new ones are described. From these numbers, around 70% of these diseases have a genetic aetiology [4]. Another problem associated with rare diseases, in addition to the lack of drugs, is the long average time of diagnosis, which can reach up to seven years. Furthermore, more than half of all rare diseases affect children, constituting a major problem for society. It is estimated that over one-third of children with a rare disease will not live more than five years, and about 35% of these children will die within the first year of life [5].

The huge number of rare diseases and the appearance of new ones every year make it extremely difficult to develop drugs due to the high costs associated with research and development processes [6]. EU legislation encourages pharmaceutical companies to develop drugs for rare diseases, so-called “orphan drugs”, because this status implies incentives for pharmaceutical companies, including 10 years of market exclusivity, protocol assistance, fee reductions for the European Medicines Agency (EMA) centralized procedures, and specific grants for orphan medicinal product (OMP) trials. However, only a small number of OMPs have been developed [7] since the Orphan Drug Act of 1983, and only 600 treatment options have been available for rare diseases [5].

One of the main reasons that hinder finding drugs for rare diseases is that developing a drug de novo is a costly and time-consuming investment, with no guarantee of obtaining an effective drug for the disease being investigated for treatment. The introduction of a new compound to the market can cost as much as USD 2.5 billion, with further increasing numbers often including high development and manufacturing costs [8]. Merely 5 out of 5000 (0.1%) experimental compounds that enter preclinical testing progress to evaluation. Only one of these five compounds receives approval from the US Food and Drug Administration (FDA) for use in humans, noting the failure susceptibility of this process [9].

In this context, it is necessary to find and develop alternatives that can reduce the time and the cost of de novo drug development. Drug repositioning is a potential alternative, consisting of identifying a new indication for existing or already-approved drugs, beyond the scope of their original use [10]. The use of drug repositioning for rare diseases has gained popularity in recent years. Repurposed drugs can reach the patient as a marketed treatment in 3–12 years [6]. They have an average cost of USD 300 million and an estimated success rate ranging from 30% to a potential 75% [11], five times more than the development of new compounds. Hence, the process of repurposing drugs for new indications, compared with the development of novel orphan drugs, is a time-saving and cost-efficient method [1].

As it has been indicated, due to the high costs, pharmaceutical companies have invested a scarce amount in the development of de novo drugs for rare diseases. Some of the principal reasons are the small number of cases in the population and the lack information available. Thus, the approximately 3000 drugs that have been approved by at least one country represent a valuable untapped resource that can be used against other diseases such as rare ones. Consequently, drug repositioning holds significant promise for the treatment of rare diseases [12].

The progress in computer science and artificial intelligence is increasing exponentially, which has favoured the research on drug-repositioning processes using computational-based techniques over the years, having developed new hypotheses through them. One of the most remarkable advances in recent years has been the development of a new methodological pathway capable of producing potential new drug-repositioning hypotheses through integrated knowledge based on biomedicine [13]. Cancer is one of the diseases that has benefited most from these studies, which outline different potential therapies to treat this disease [14,15].

Nevertheless, the disease that has stood out par excellence in the field of drug repositioning has been COVID-19. In the last two years, many scientific papers have been published based on these ideas [16,17,18]. Within COVID-19, we can emphasize a study based on the creation of different information paths to find 13 potential drugs to treat the symptoms of COVID-19 and targeting SARS-CoV-2-related genes [19]. In the work by Gysi et al. [17], deployed algorithms relying on artificial intelligence, network diffusion, and network proximity, tasking each of them to rank drugs for their expected efficacy against SARS-CoV-2.

Studies based on drug repositioning in rare diseases are scarcer. This is mainly due to the small number of people affected, which makes it more difficult to obtain information on these diseases. Despite this problem, one of the most relevant studies found in the scientific literature is based on the discovery of potential new treatments for adrenocortical carcinoma (ACC) through a model named Heter-LP that identifies innovative putative drug–disease, drug–target, and disease–target relationships for ACC [20]. Additionally, noteworthy are the studies focused on finding a possible treatment for Alzheimer’s disease based mostly on consensus methodologies and reviews focusing on prioritisation processes [21,22]. Another study was based on the use of networks (SAveRUNNER) for drug repositioning in amyotrophic lateral sclerosis disease [23].

Since, as mentioned above, studies based on rare diseases are very hard to find, the creation of new computational methods for the discovery of potential treatments for these diseases is essential. Thus, this will be the main objective of the study that we present in the following sections. In this work, we propose a method for the identification of potential drug-repurposing hypotheses in rare diseases by using the data available at the DISNET (https://disnet.ctb.upm.es (accessed on 8 May 2021)) platform [24]. DISNET contains information of symptoms, genes, drugs, protein interactions, among other features, related to more than 24,000 diseases. In addition, it contains the relationships that exist between these features and the diseases, which is a great driving force for the prioritisation of new repurposing cases. Different computational drug-repositioning methods have been developed to find potential treatments for rare diseases. These drugs will be further validated in the scientific literature. Furthermore, specific information about rare diseases has been collected from the Orphanet database in order to obtain the global prevalence and the prevalence value of these diseases.

## 2. Materials and Methods

In this section, the materials and methods carried out throughout the study will be explained, differentiating two important subsections. On the one hand, materials, where the data acquisition and integration processes will be described. Furthermore, the selection of the rare diseases that have been considered in this work will be discussed. On the other hand, methods, focused on the explanation of the different drug-repositioning computational methods that have been developed. The validation procedure of the potential drugs and their classification according to the ATC (https://www.who.int/tools/atc-ddd-toolkit/atc-classification (accessed on 19 May 2022)) will be defined. Consecutively, we will turn our attention to the phenotypical similarity comparison between the rare and non-rare diseases. Finally, the disease–gene associations between the genes of the rare diseases and the genes that encode the targets of the potential drugs will be analysed.

### 2.1. Materials

#### 2.1.1. Data Acquisition and Integration

The integration and acquisition of the data for this study have been carried out using, mainly, two data sources. The first source of data was DISNET. DISNET is a database that collects relevant biological information about diverse pathologies by extracting such data in a textual and structured format from public sources [24]. This platform consists of three different layers: the biological layer (containing diseases’ associations to genes and proteins, among others), the phenotypical layer (containing primarily disease–symptoms associations), and the drugs layer (which stores drug-related data, including their associations to diseases and the drugs targets). From this platform, the genes and symptoms associated with the rare diseases considered for the study were retrieved. Since DISNET updates its data on a regular basis (phenotypical data from at least two sources are updated every 15 days), it is necessary to specify that the extraction of the data that will be used in this analysis was carried out on 8 May 2021.

The second one was Orphanet, which is a network of 37 countries, co-founded by the European Commission, whose aim is to increase knowledge about rare diseases to improve the diagnosis, care, and treatment of people with these diseases [4]. This database collects information on treatments, clinical trials, and prevalence, among many other data, for all rare diseases existing to date. The relevant information on this database for the study performed on this work was the rare disease name, geographical prevalence, and its associated prevalence value.

The epidemiological data were obtained from XML epidemiological data in Orphadata. They contained a total of 6043 diseases with 15,439 prevalence records associated with the diseases, as the same disease can have different prevalent values in different countries or areas of the world.

**Figure 1:**
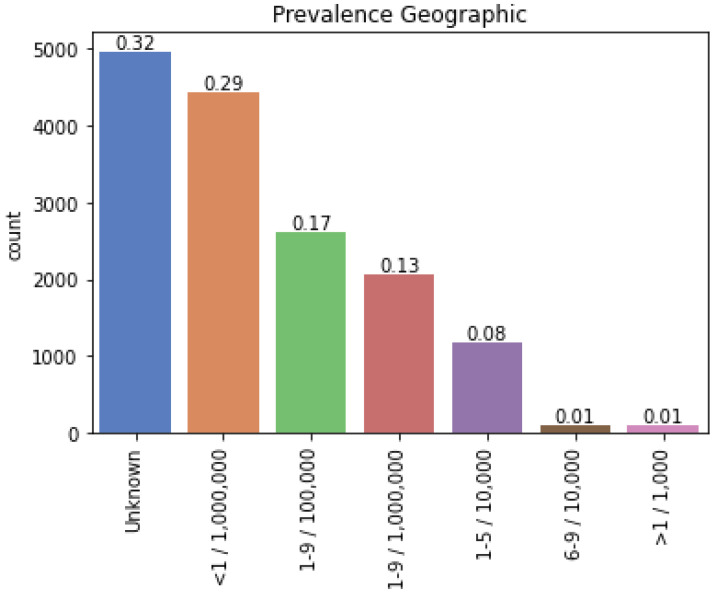
Representation of the distribution of the different prevalence values in the set of rare diseases present in Orphanet.

The prevalence value of diseases according to the criteria used in Orphanet is divided into nine groups. Within these groups, three refer to unknown data (Null, Unknown, and Not yet documented) and which are grouped into a single group for the graphical representation (Figure 1) of the prevalence values found in the rare diseases present in this database. The other six groups present, ordered from highest to lowest prevalence are: >1/1000, 6–9/10,000, 1–5/10,000, 1–9/100,000, 1–9/1,000,000 and <1/1,000,000. The classification of diseases according to geographical prevalence is distributed into 133 different locations where global prevalence and division by continent stand out.

A total of 6043 rare diseases were collected from the Orphanet database. However, the number of those diseases presented in DISNET were only 3785. On the other hand, a crucial part of this study was to filter those diseases that did not have an associated treatment on this platform. Eventually, the last requisite was that all the pathologies had information related to genes and symptoms in DISNET. Applying all the requirements, the total number of rare diseases considered was 519. Data collection and analyses carried out throughout this study were carried out using the Python tool, and the code is available online(https://medal.ctb.upm.es/internal/gitlab/b.otero/computational_aproaches_dr_rare_diseases (accessed on 13 July 2022)).

#### 2.1.2. Selection of Rare Diseases

As it has been mentioned in the filter phase explained above, the number of diseases which meets the appropriate criteria for this study comprises 519 diseases. Since this number is still too high for a detailed analysis, an additional filtering was carried out to select a restricted population. Hence, the definitive set of disorders was selected following four major criteria: (i) how many genes were associated, (ii) how many symptoms, (iii) the geographical prevalence, and (iv) the prevalence value itself.

**Figure 2:**
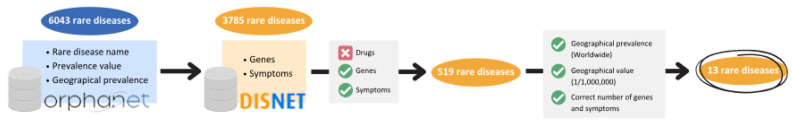
Workflow followed to choose 13 rare diseases to be studied.

We decided to focus on rare diseases with a global prevalence to achieve results with significant societal–scientific outcomes. Furthermore, finding treatments for excessively rare pathologies, that is a prevalence value of 1/1,000,000, would be a far more important discovery since it is a more arduous task. Finally, rare diseases should have a proper value of genes and symptoms from a computational cost perspective (Figure 2).

After all, a definitive list of 13 rare diseases was selected to perform the experiments. Table 1 specifies their name and Unified Medical System Language (UMLS) Concept Unique Identifier (CUI) (https://www.nlm.nih.gov/research/umls/index.html (accessed on 25 May 2021)) and their number of associated genes and symptoms. The disease CUI allows the normalization of the data for its query in the different databases that are used on this analysis.

### 2.2. Methods

#### 2.2.1. Drug Repositioning

**Figure 3:**
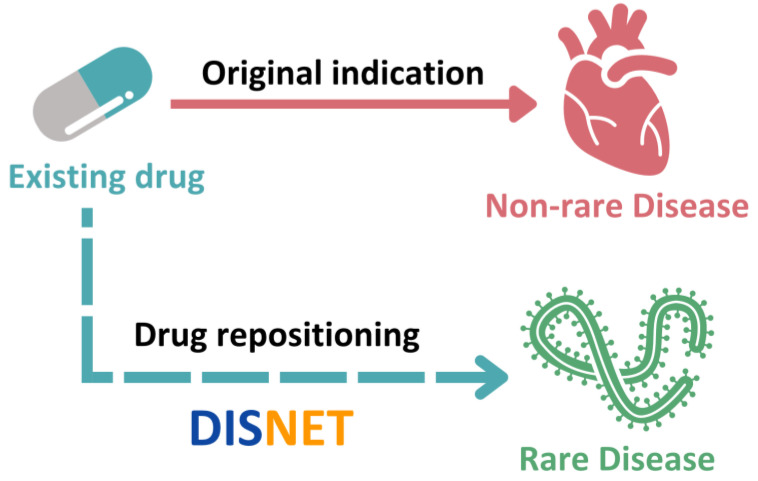
Description of the methodological background of the main objective of the study.

Advanced biomedical data are harnessed to identify new indications for existing drugs using computational drug-repositioning techniques. A graphical summary of the objective to be achieved in this study is shown in Figure 3. Based on an existing drug with an original indication for a non-rare disease (in this case, a heart disease), the aim is to use the data available on the DISNET platform to find a drug-repositioning hypothesis for a rare disease (in the example, Ebola disease).

**Figure 4:**
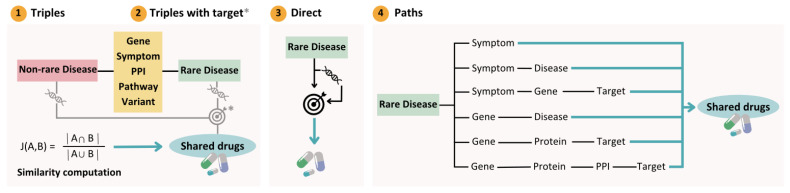
Summary of the four computational methodologies applied to obtain drug repositioning in rare diseases.

Four computational methods were proposed (see Figure 4) to obtain drug candidates to treat the 13 rare diseases previously mentioned: (1) triples approach, (2) triples with associated target approach, (3) direct approach, and (4) paths approach.

**(1)**  **Triples approach.** The first strategy was to use triples for drug repositioning. A rare and a non-rare disease can be associated by a biological feature. The similarity between them through a specific feature is called triples. In this work, five kinds of triples were designed based upon five fundamental biological factors:
  - Non-rare disease–Gene–Rare disease;
  - Non-rare disease–Symptom–Rare disease;
  - Non-rare disease–Protein interaction–Rare disease;
  - Non-rare disease–Pathway–Rare disease;
  - Non-rare disease–Variant–Rare disease.

To compute the biological feature-based similarity between non-rare and rare diseases, we used Equation (1).

(1) $$Jaccard\;\left(A,B\right)=\frac{|A⋂B|}{\left|A⋃B\right|}$$

To begin with, each of the rare diseases in the definitive set was matched with all the non-rare diseases present in the DISNET database. On the basis of the five biological characteristics, a similarity score was computed for all the pairs of “non-rare disease—rare disease”. Biological properties were ranked from the highest to the lowest similarity scores. Afterwards, in each set of biological characteristics, the five main non-rare diseases associated with the specific rare disease were chosen. Thus, we had the top 5 non-rare diseases derived from the similarities of genes, symptoms, protein–protein interaction, pathways and variants for individual rare diseases. The subsequent process was to obtain the drugs related to the top non-rare diseases.

To establish candidate drugs for repositioning to treat the related rare disease, we looked for drugs that were shared among those linked to the top 5 non-rare diseases in their respective biological collection. Our next step was to determine which drugs appeared in all feature groups (set intersection). We considered that these drugs could be potentially repositioned as treatments for the rare diseases in the study based on two ideas: (1) the selected top 5 pairs had the highest biological feature based similarity score, showing a clear similarity between the rare disease and the non-rare disease; and (2) the intersection among the groups of those characteristics allowed us to find only those disease pairs where the relationships were based on several characteristics, reinforcing the idea that those pairs were having potential shared underlying molecular elements or interactions.

**(2)**  **Triples with Associated Target Approach**. In this case, the triples are composed by the rare and non-rare diseases, and one biomedical feature. The difference with respect the previous method was that we forced the two diseases to share the gene that encoded the protein target of the non-rare-disease-associated drug. Then, the drug detection procedure was carried out as described in the above approach.
**(3)**  **Direct Approach.** An encoding gene for a drug target was associated with the rare disease. As a result, the drug can be used to treat rare diseases because there is a direct relation between the disease and the drug.
**(4)**  **Paths Approach.** This method is based on creating 6 strategies, named as paths, following the diseases’ biological characteristics and associated drugs. The drugs intersecting in these 6 paths (excluding those returning empty sets) are considered as the final list of this fourth computational approach.
  - Rare disease–symptom–drug: we obtained the drugs which are indicated for the symptoms of the rare disease.
  - Rare disease–symptom–disease–drug: we selected the drugs associated with the diseases that shared symptoms with the rare disease.
  - Rare disease–symptom–gene–target–drug: given a rare disease and its symptoms, those non-rare diseases that present the same symptoms were searched. From these symptomatically similar diseases, we extracted their genes, targets and related drugs.
  - Rare disease–gene–disease–drug: we obtained the drugs used for the diseases that shared genes with the rare disease.
  - Rare disease–gene–protein–target–drug: in order to obtain the potential drugs, we obtained the genes, proteins and targets associated with these rare diseases.
  - Rare disease–gene–protein–protein interaction–target–drug: from all of the rare diseases, we extracted the genes, proteins and the powerful protein–protein interactions data. After having obtained this information, we collected the associated target from which the corresponding drug.

#### 2.2.2. Validation in Scientific Literature and Clinical Trials

The drugs obtained through the explained computational methods for the rare diseases under study needed to be corroborated. For this aim, an exhaustive search was carried out from a literature and clinical trials standpoint. We checked information present in platforms such as PubMed (https://pubmed.ncbi.nlm.nih.gov (accessed on 11 June 2021)), scientific journals, and the clinical trials website (https://www.clinicaltrials.gov (accessed on 11 June 2021)).

The process of verifying the repositioning of candidate drugs was carried out following steps:

1. The names of the drugs and diseases were searched on Pubmed, Google, and clinicaltrials.gov.
2. We checked the presence of results that related to both concepts.
3. If positive results were obtained, all were selected. If the number was very high, the first 10 were selected.
4. The chosen information was read.
5. A conclusion was drawn for each relationship: whether the drug treats the studied disease or, on the contrary, causes it.

We found cases with a large amount of information linking the disease being studied to the potential treatment. If this occurred, the first searches that appeared in the information source were selected. There were also cases in which the information was very scarce, and the references obtained were all those found. However, there are also many drugs for which no information was found relating the treatments to the rare diseases studied.

The set of potential drugs that were computationally obtained and that have been verified with the scientific literature or clinical trials will be referred to throughout the paper as “checked-drugs”, to facilitate their reference in the text.

#### 2.2.3. Drug Classification

Once the checked-drugs set was identified, we extracted their classification to analyse the nature of the drugs in search of patterns. That is, to see if any categories are more susceptible to repositioning than others.

To classify the drugs, the categories established by ATC were considered. Based on this classification of drugs, the different groups are formed considering the organ or system that will be affected by the drug, as well as its pharmacological, therapeutic, and chemical properties. Depending on these characteristics, five levels are obtained:

- ATC 1st level: Anatomical or pharmacological groups.
- ATC 2nd level: Pharmacological or therapeutic subgroup.
- ATC 3rd and 4th levels: Chemical, pharmacological, or therapeutic subgroup.
- ATC 5th level: Chemical substance.

The level to be borne in mind in this work for the classification of potential candidates is going to be ATC 1st level.

#### 2.2.4. Phenotypical Similarity

For drug repositioning, the fact that two diseases share many symptoms can be a good starting point to find a new target for an existing drug. For this reason, part of this study focuses on testing the phenotypic similarity (PS) of rare diseases for which drug repositioning has been found both computationally and bibliographically, with the non-rare diseases that were the original indication for those drugs. The only condition was that both diseases (non-rare and rare) shared the gene that encoded the target of the corresponding drug.

The phenotypical similarity was calculated using the Jaccard’s index, as explained in the previous Section 2.2.2. On the one hand, the similarity was computed between the rare diseases and the non-rare diseases with which they shared the gene that encoded the target protein of the corresponding drugs. On the other hand, the similarity was calculated for the rare diseases and the rest of non-rare diseases present in DISNET database. The comparison between the two datasets was made using a Welch’s T-test to determine if there were statistically significant differences between the two groups. The idea behind this experiment was to verify whether the phenotypical connections between the non-rare and rare diseases in the newly generated repurposing hypotheses were stronger than the rest of non-rare–rare disease pairs in DISNET.

#### 2.2.5. Disease–Gene Associations (GDAs)

As part of this study, we aim to use for validation purposes the relationship between the rare disease and the target gene of the potential drug proposed to treat this disease. We only considered the checked-drugs dataset. This relationship is measured using the gene–disease score present in the DISNET database, previously extracted from DisGeNET, which is an in-house metric varying from 0 to 1 and that represents how well established a particular association is based on the current knowledge.

DisGeNET is a digital discovery platform hosting one of the greatest public collections of genes and variants associated with human pathologies. The update version of DisGeNET (v7.0) includes 1,134,942 gene–disease associations (GDAs), between 21,671 genes and 30,170 diseases, disorders, traits, and clinical or abnormal human phenotypes, and 369,554 variant-disease associations, between 194,515 variants and 14,155 diseases, traits, and phenotypes. In the DISNET platform, a total amount of 358.209 GDAs are collected, coming from the above-mentioned DisGeNET [18].

In addition to the GDA score, we obtained the disease specificity index (DSI) that varies from 0 to 1 and is inversely proportional to the number of diseases associated with a particular gene. Furthermore, the disease pleiotropy index (DPI) was included, which varies from 0 to 1 and is proportional to the number of different disease classes with which a gene is associated.

Finally, once the value of this association had been obtained using the GDA score, we checked, for each of the rare diseases, whether there was a greater association between the target gene and the rare disease than between all the DISNET diseases and their respective genes. For this purpose, the Mann–Whitney U Test was used since the data did not follow a normal distribution.

## 3. Results

### 3.1. Drug Repositioning

We have generated drug-repositioning hypotheses in the scope of rare diseases. The 13 diseases previously selected have been studied applying the aforementioned four computational methods individually.

In Table 2, we represent the results obtained from each approach, as well as the final result that arose from the intersection of the non-empty sets. That is, we searched those drugs that were in common in all the approaches in which at least one possible candidate drug was obtained. Two different columns refer to the final results. On the one hand, ALL represents the intersection of the non-empty sets of all approaches. On the other hand, the column TT.DDR.P represents the intersection without the Triples approach since it is too generalist.

We found potential drugs for the 13 rare diseases. However, the Lown–Ganong–Levine syndrome disease was discarded because the number of drugs obtained was very high (965), and they were very non-specific. The reason for this result is that, for this disease, potential drugs were only obtained by one of the designed methods (paths) instead of several like the others. In addition, the potential drugs had very general uses.

### 3.2. Validation in Clinical Trials

Upon the candidate drugs for repositioning which were obtained using the proposed methods, it was necessary to check whether these drugs had been considered in the scientific literature to treat the rare disease that is being proposed as a new treatment.

Out of the 378 drugs that we obtained computationally as possible drugs to be repositioned, we found in the scientific literature 60 drugs (checked-drugs) that have been validated as possible candidates to treat the rare diseases under study. From these 60 drugs found in the scientific literature to be related to the rare diseases under study, 33 of them were identified as toxic, that is, they produce the disease or promote its occurrence. Nonetheless, a total of 27 drugs have been obtained that can treat the symptoms caused by the particular disease or prevent complications that may result from the disease (Table 3). These drugs, which have finally been obtained for the set of rare diseases selected for this research, do not correspond to treatments that can end these diseases, but the term treatment is used because they could help patients suffering from these pathologies to have fewer complications in their daily lives. When we talk about treating a disease, it is not synonymous with cure, but in many cases, what is achieved with treatments is to improve or extend the life of patients and these 27 drugs can be a great starting point.

We describe, hereunder, the potential scientific justifications described in the literature supporting the presented hypotheses.

Dejerine–Sottas syndrome. The two drugs (dexamethasone and thalidomide) that were obtained to be repurposed were found in the scientific literature for Charcot–Marie–Tooth (CMT), Derejine–Sottas being a specific type of CMT.

- Dexamethasone. It is used for the treatment of CMT disease [25].
- Thalidomide. It is potentially toxic for patients with CMT [26]. In high doses, it increases the risk of peripheral neuropathy [27,28].

Locked-in syndrome. A total of 80 drugs were obtained computationally and 11 of them were found in the literature. Furthermore, most of them were toxic, that is, the cause of the disease.

- Amphetamine. Applying stimulant drugs such as this can help brain communication occur when the patient has this disease [29].
- Baclofen. Unsatisfactory data are obtained in patients treated with this drug presenting locked-in syndrome [30]. Another study was found where the data obtained are not conclusive [31].
- Cisplatin together with doxorubicin produces the disease in combination with intrathecal cytosine arabinoside or methotrexate, which is another of the drugs that has been found for repositioning [32].
- Cytarabine. Another of the drugs obtained, cytarabine, is related to this line [33].
- Cocaine. A clinical case was collected where a woman developed this disease after the abusive consumption of cocaine [34].
- Deferoxamine. It is a risk factor for this disease when used for iron chelation [35].
- Furosemide. It produced locked-in syndrome in a case study when furosemide was being used to treat anasarca disease [36].
- Methamphetamine. The abuse of this substance can lead to neurological diseases that trigger locked-in syndrome [37].
- Methylprednisolone. It has been suggested to treat paraneoplastic symptoms but to a limited extent, because it is highly toxic [30].

Diffuse cutaneous mastocytosis. The four drugs that were obtained by the computational methods were found in the scientific literature.

- Alcohol. In accordance with the literature recommendation, patients with this diagnosis should avoid taking it [38].
- Silver sulfadiazine. A skin treatment to be applied on erosion areas affected by this pathology [39].
- Dexamethasone. Corticosteroids and glucocorticoids, to which this drug belongs, are used to palliate the symptoms of this pathology [39,40,41,42].
- Acetaminophen. Postoperative patients used it to relieve pain [40,42].

Congenital neuronal ceroid lipofuscinosis. A total of 48 possible drugs for this disease were obtained. Of the 48, 7 drugs have been found in the scientific literature.

- Carbamazepine. It is stated that it should be avoided in conjunction with other sodium channel blockers because it can increase seizures despite being an anticonvulsant drug [43].
- Copper. The CNL6 gene participates in this disease. When the CNL6 gene is impaired, it produces the accumulation of biometals such as copper and this leads to the pathogenesis of the CNL6 disease. More research is needed on the function of the CNL6 gene [44]. Therefore, copper would not be an effective treatment against this disease.
- Dexamethasone. Its efficacy has not been proven 100%, more studies are needed to see if corticosteroids could influence the progression of the disease of the CNL3 gene by decreasing it [45]. What we hypothesize is that this anti-inflammatory treatment with corticosteroids may be beneficial in ameliorating some of the symptoms of juvenile CLN3 disease [45]. It also serves to alleviate the symptoms of another treatment that has been tried in this disease [46].
- Gentamicin sulfate. Literature evidence has been found for gentamicin. It can read premature termination codons (PTCs) and partially restore protein expression or function. PTC mutations are present in the CLN2 type of disease, and gentamicin can carry out this restoration [45].
- Methionine. It increases vacuolar acidification, which elevates the useful life of the vacuole. This mechanism affects a metabolic pathway in yeast proven for this disease [47].
- Valproic acid. It is effective in controlling seizures in this disease [43].
- Tamoxifen. It increases the production of cathepsin D, which helps to prevent this disease from occurring since it has been shown that many neurodegenerative diseases arise when there are low levels of cathepsin D or it is inactive, causing failures in one of the genes that produces this disease [48].

Schwartz–Jampel syndrome. A total of 14 drugs were obtained but only information about one of them was found in the scientific literature.

- Carvedilol. It is associated with this disease through congenital myotonia. It is used for heart problems, some of them produced by congenital myotonia [49].

Seckel syndrome. Two drugs were obtained from the repositioning approaches, but only one was obtained in the literature.

- Caffeine. This disease occurs as a response to DNA damage in the replication fork. Caffeine inhibits the activity of ATR kinase, which prevents DNA damage from being repaired; therefore, this molecule favours the appearance of this syndrome [50,51].

Neonatal hemochromatosis. A total of 91 potential drugs were obtained through computational methods. Out of these 91, only 8 have been found in the scientific literature. Within these eight, five are possible causes of the disease and three can be considered as treatments for liver problems.

- Acetaminophen. It promotes the accumulation of excess iron in the liver leading to the appearance of the disease under study [52,53].
- Albendazole and fluconazole. Both drugs cause liver failure and can, therefore, trigger liver failure [54].
- Alcohol. It results in hereditary hemochromatosis in foetuses [55,56].
- Ceftriaxone. It causes neonatal liver pathologies such as the one studied [57].
- Ciprofloxacin, sulfamethoxazole, and doxycycline. They are used to treat infections associated with haemochromatosis [58].

In addition, within the 91 drugs, 10 more drugs were found to be related to the treatment of certain liver problems, such as liver cancer. Additionally, some of them cause different liver diseases.

X-linked lymphoproliferative disorder. The drug obtained through repositioning routes was not present in the scientific literature associated with this disease. Therefore, we could not conclude that it was a potentially repositionable drug for this disease.

X-linked Emery–Dreifuss muscular dystrophy. A total of 126 drugs were obtained by computational repositioning methods. Of these 126 drugs, 15 were found in the literature, and 11 can treat the disease.

- Amiodarone. It is used to treat or prevent heart failure in patients with this disease [59,60,61].
- Aspirin. It is used as a prophylactic treatment to prevent thromboembolism in patients with this disease, but its efficacy has not been fully demonstrated, and more studies are needed [62].
- Carbamazepine. It is used to treat the epileptic seizures that occur in this disease and other muscular dystrophies [63,64].
- Cyclosporine. It is used to perform immunosuppression that could be useful to perform heart transplants in patients with Emery–Dreifuss Muscular Dystrophy (EDMD) [59].
- Dantrolene. Anesthetic that was previously used for muscular dystrophies and may pose a risk to some patients [65].
- Enalapril, losartan, and enalaprilat. Both drugs are ACE inhibitors that are the first line of treatment for chronic heart failure although a substitute combination is being sought in this study [61]. In another article, this treatment is found to be used for heart failure [66]. They also help the blood vessels open wider and the heart can pump blood with less pressure [67].
- Isoproterenol. Atrial arrhythmias are a major problem in EDMD disease. Isoproterenol is used to prevent blockages in the heart or cardiac arrest, in a similar manner to epinephrine. In this study, it is administered to the patient at high levels but without causing arrhythmias [68].
- Methamphetamine. It produces dilated cardiomyopathy, as cocaine and amphetamine do [61,69].
- Metoprolol. It is indicated to control the heart rate and prevent further arrhythmias [68].
- Sirolimus. Rapamycin is a synonym, and it is used to prevent the progression of cardiomyopathies in mice. With it, a metabolic remodelling has been reached, which could be giving rise to a cardioprotective mechanism that slows the progression of EDMD and improves its prognosis [70].
- Valproic acid. It is used to treat the epileptic seizures that occur in this disease and other muscular dystrophies [63].

Moreover, other drugs obtained from the computational approaches were found in the scientific literature, not directly indicated for this disease but related with other associated diseases or symptoms. Amlodipine, propranolol, and verapamil are treatments for cardiac problems, but they did not appear as such in the literature in relation to the disease. Propranolol has a molecular structure like isoprenaline, which is the drug found in the scientific literature to treat heart problems. This is because both are beta-adrenergic, but one is an agonist and other one is an antagonist of these receptors. Dexamethasone and gentamicin sulfate are used for Duchenne muscular dystrophy, which has conditions in common with this disease, so they could probably be repositioned for both diseases. Phenobarbital is used for the treatment of epilepsy, which is one of the symptoms that appear in other diseases related to muscular dystrophy but is not the case in the disease we are considering. Zoledronic acid is used for laminopathies which is a disease genetically related to the disease under study. Ramipril is another ACE inhibitor; it does not appear as such in the scientific literature, but it has the same molecular structure as the other two ACE inhibitors that have been found by computational repositioning approaches and have been justified with the literature (enalapril and enalaprilat). It appears in the section of similar structures in DrugBank.

Dahlberg–Borer–Newcomer syndrome. Eight drugs were obtained from the computational repositioning approaches, but none of them were found in the literature. However, the eight drugs have in common that they are kinase inhibitors. These drugs cause thyroid failure, one of the consequences that occurs in this disease, hypoparathyroidism [71].

Vibratory urticaria. The two drugs that were obtained computationally as repositionable drugs are present in the scientific literature.

- Valproic acid. It causes skin reactions along with other drugs used to treat nervous system problems [72,73].
- Estradiol. It promotes mast cell release by causing increased histamine liberation resulting in hives/chronic urticaria (seen in women taking contraceptives or hormone replacement therapy) [74].

Acromegaloid facial appearance syndrome. The drugs found for this disease in the scientific literature were indicated for general acromegaloid. Of the two drugs obtained, only one of them was found in the literature. Glyburide was found under the name Glibenclamide. Patients with acromegaloid have increased growth hormone and insulin resistance, which makes them insulin-dependent. Glibenclamide is used to treat diabetes [75,76].

### 3.3. Drug Classification

The drugs obtained as potential treatments for the rare diseases studied were classified according to the ATC code. A total of 60 drugs were considered for this part of the study, which are listed in the checked-drugs set.

**Figure 5:**
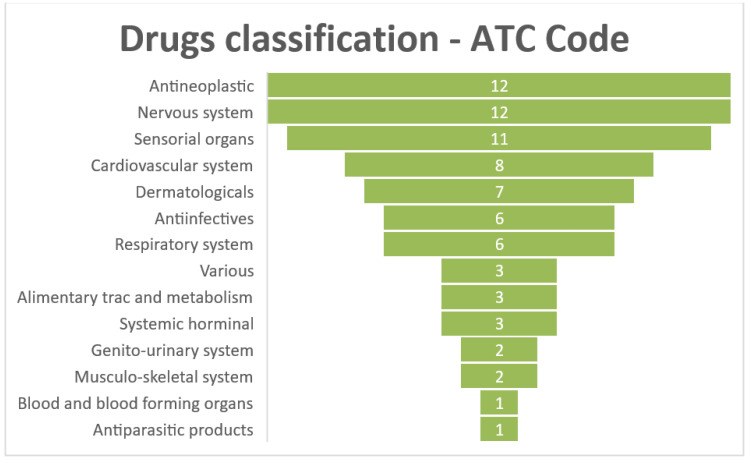
Classification of the potential drugs according to the first level of the ATC code.

As Figure 5 shows, there are two categories (antineoplastic and immunomodulating agents, and nervous system) that stand out in the classification of drugs according to the ATC code.

It should also be noted that within the set of 60 drugs, we find the presence of at least one of them in all the possible 14 categories that make up the first level of ATC code classification. This is a very important fact in the results obtained in this research since it indicates that there is great heterogeneity among the drugs proposed as potential candidates for repositioning, and this may be due to the complementarity of the computational RD methods proposed in this study. This means that the developed methods can find possible treatments for the diseases without biasing by the type of drugs they are; they are all considered equally.

### 3.4. Phenotypical Similarity

PS between the (i) rare–non-rare diseases involved in the repurposing hypotheses compared to (ii) the disease pairs in the DISNET database varied significantly according to these two groups of diseases considered.

Five rare diseases for which potential repositionable drugs were obtained through computational methods and in the scientific literature were considered for this comparison.

In Table 4, we can observe the results obtained by comparing rare and non-rare diseases by their symptoms.

To sum up, Dejerine–Sottas syndrome, Schwartz–Jampel syndrome, and Dahlberg–Borer–Newcomer syndrome presented a phenotypic similarity with the non-rare diseases (in their respective repurposing hypotheses) above the mean of DISNET disease pairs, showing statistically significant differences. However, in the case of Acromegaloid facial appearance syndrome and Seckel syndrome, the phenotypic similarity with the non-rare diseases was below the mean of DISNET disease pairs (showing here statistically significant differences as well).

### 3.5. Disease–Gene Associations (GDAs)

Out of the 13 rare diseases considered for this study, the gene–disease association could be obtained for 5 of them. This is due to, as explained above, the fact that we only used those diseases with computational drug-repositioning results and that shared the gene that encoded the target of the potential drugs. Additionally, these drugs were related to the rare diseases in the scientific literature.

**Figure 6:**
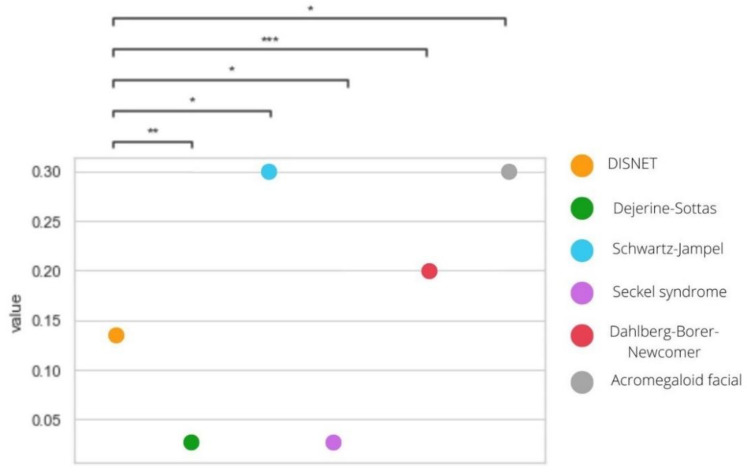
Representation of the statistical differences between the GDA score value of the rare diseases considered and the DISNET value. *p*-value annotation legend: ns: 5.00 × 10^2^ < *p* < = 1, *: 1.00 × 10^−2^ < *p* < = 5.00 × 10^−2^, **: 1.00 × 10^−3^ < *p* < = 1.00 × 10^−2^ and ***: 1.00 × 10^−4^ < *p* < = 1.00 × 10^−3^.

In Figure 6, we can see the values of GDAs scores for each of the rare disease–gene involved in the repurposing hypothesis pairs statistically compared with the value of the rest of GDA scores in DISNET database.

In this representation, statistically significant differences between all the studied rare diseases compared to the mean obtained from DISNET can be observed. Although all of them present such differences, in the case of the rare diseases Dejerine–Sottas syndrome and Seckel syndrome, these differences are reversed from what we expected. That is, the GDA score value is higher in the case of the DISNET database. For the rest of the rare diseases, we can see that GDAs are stronger between the potential drug target gene and the rare disease than in rest of the GDAs present in DISNET.

In Table 5, we can observe the results obtained for these rare diseases and their associated target genes. The DSI and DPI values are also shown.

## 4. Discussion

The use of drug repositioning is a potential field of study to find new treatments for rare diseases in a shorter period. The development of this type of studies can have great social benefits since medicines can be found for diseases that are not being as thoroughly investigated and this can cure or alleviate the symptoms of many people suffering from these pathologies. Rare diseases affect many people worldwide. In addition, new ones appear every year, increasing the great diversity of rare diseases that exist today. The fact that there are so many of them and that separately, they affect a small number of people, makes it much more complicated to investigate them. Despite this, this study has been able to verify that the use of drug-repositioning techniques for rare diseases is a useful and effective strategy for finding treatments.

The DISNET platform used throughout the study is proposed as a tool and potential data source for obtaining treatments for rare diseases in conjunction with the computational techniques developed throughout this research. DISNET can be established as a starting point in the search for new treatments for diseases, since, although in this study we have focused on rare diseases, this platform has been verified in other research as a useful tool for drug repositioning, such as for COVID-19.

In this work, we have proposed a series of repurposing hypotheses for 13 different rare diseases. We have followed four computational methods that involve rare disease-related features including genes, protein interactions, pathways, symptoms, and drug targets. Through different approaches considering these connections we obtained different drugs with potential of treating each disease. We here discuss the most important findings.

The resulting drugs have been verified in the scientific literature. In 75% of the cases, drugs were found to be potentially therapeutic, although evidence was also obtained for 92% of them. If we consider the total number of drugs included in this 92%, we would also be including references that point to purely toxic treatments. Even so, it is an interesting value to consider, because it shows that there are underlying characteristics of those that have been analysed that represent relationships between diseases, although these are not always necessarily positive. Nonetheless, the data obtained are very favourable considering the difficulty of finding scientific literature dealing with these rare diseases.

Within this section, it is of added value to focus on the rare disease **Schwartz–Jampel syndrome**, which is a neuromuscular disease of genetic origin that mainly affects neonates. For this disease, 14 potential drugs have been obtained by computational methods and one of them has been verified in the scientific literature. The drug Carvedilol belongs to the type of cardiovascular system within the ATC code classification used in this work. This type of drug is in the top five of the most common drug types we obtained in our case. If we focus on the phenotypic similarity, even though this rare disease has only a total of five associated symptoms, it shows a very high similarity value (0.84) with the selected non-rare diseases. Additionally, therefore, it strongly diverges from the similarity value obtained in DISNET showing statistically significant differences. As for the GDAs, this disease has an association score value with VEGFA gene of 0.3, which is not a very high value since the maximum is 1, but it is the highest value we have obtained in this study. Furthermore, it shows statistically significant differences when compared with the mean value obtained in DISNET. The associated DSI value is low, so the gene is associated with a high number of diseases and is therefore not very specific. The DPI value is large, so this gene is usually associated with the same type of disease.

Of the potential drugs also found in the scientific literature, a classification has been made according to the ATC code, where most of the drugs obtained as possible treatments for these rare diseases are divided into two categories: antineoplastic and immunomodulating agents, and nervous system. It is worth noting the importance of antineoplastic drugs used for cancer treatment, which are based on inhibiting the production of proteins that prevent the immune system from fighting cancer cells. Of the rare diseases studied, none of them are representing carcinoma. However, due to the great deregulation that occurs in the human body when suffering from cancer, since it is a disease that affects many biological processes, these drugs can target many pathways and could be potential drugs to treat a specific rare disease. Therefore, a large number of potential drugs are drawn from this category. It should also be noted that research into this type of drug is enormous, so there is a wide range of options that could be reused for the treatment of rare diseases since, as this study is demonstrating, these drugs play a very important role. A specific case of the rare disease Dahlberg–Borer–Newcomer syndrome is that seven antineoplastic drugs (see Table 5) out of the nine verified in the scientific literature have been obtained to treat this disease. It is a dysmorphic/multiple congenital anomaly syndrome in which many of the biological pathways that are involved in the development of this disease may be affected by failures that correct or prevent these drugs. Another important type of drug is those of the nervous system; of the 13 rare diseases studied, 6 of them are classified as diseases of the nervous system. For this reason, it is understandable that most of the drugs that have been found as potential treatments for them belong to this category.

Even so, at least one drug has been obtained for each of the categories considered by this classification. This demonstrates the variability present among potential drugs and how our model can find future treatments without being biased by the category to which they belong. Furthermore, it allows a generalization of the model used because it is not only effective for finding a specific type of drug but also for all existing categories of ATC. Knowing which the predominant categories in the potential repositioning cases for rare diseases are will allow us in the future to narrow down the search for drugs as possible repositionable treatments within these categories. This would mean considering that it is more likely to find a potential treatment for rare diseases in these categories.

If we look at the phenotypic similarity, we can conclude that the importance of this measure in drug repositioning is strictly related to the type of diseases being compared. The diseases considered in this study show very different patterns. Within our data, we found that three of the three cases studied support the known hypothesis that phenotypic similarity is an important feature in the selection of a drug candidate, as there are numerous cases of drugs being used for different diseases only because they share symptoms.

However, we found two cases that did not follow this line, showing a lower phenotypic similarity than the one found in DISNET. This happens in the diseases acromegaloid facial appearance syndrome and Seckel syndrome. In the case of acromegaloid facial appearance syndrome, it has 90 symptoms associated. The problem is, in this case, that these symptoms are very disparate, which makes it more complicated to find shared phenotypic features, since some of these symptoms may coincide with some diseases, but not in such a high number that a high similarity value can be found. In the case of Seckel syndrome, only four symptoms are associated with it. All of them are very specific symptoms that are related to a few diseases. Two of these symptoms, “Cryptorchidism” and “Low birth weight” are related to less than 165 diseases. This represents a very small percentage compared to many existing diseases. This may explain why the phenotypic similarity found for this disease was so small.

The GDA scores studied have shown favourable results for most of the rare diseases considered. Based on these results, we can consider this GDA score as a good measure for the selection of drug candidates. However, it should also be noted that for two of the diseases studied, the expected results were not obtained. Seckel syndrome has the ATM gene related to its potential drug target. This gene does not play a major role in the disease as it is mainly caused by faults in three genes of the SCKL family. This may explain the low degree of association between the disease and the gene. In the case of Dejerine–Sottas syndrome, the three related genes, as in the previous case, are also not part of the major mutations that give rise to this disease. One of the related genes (TNF) is a very common gene in many diseases because it is the tumour necrosis factor. It has a DPI close to 0.97 so it is present in all types of diseases, and a very low DSI because it is very unspecific. These are some of the factors that may explain why there is no strong association between these genes and the rare disease studied.

## 5. Conclusions

Rare diseases are pathologies that affect a small percentage of the population. Accordingly, research and treatments for them are limited. Drug repositioning is the process of finding new therapeutic purposes for existing drugs. In this work, we have suggested four different computational approaches to provide new drug-repositioning hypotheses, opening valuable prospects in the scope of rare diseases. As the **main conclusion**, we can state that given the scarcity of treatments for this type of disease, data-driven methodologies can help in finding already-existing medicines to treat rare diseases. Focusing on the results obtained, we can conclude that the computational methods developed to suggest treatments for the selected rare diseases are effective and useful, since repositioning candidates have been obtained for the 13 diseases. This opens the door to applying this methodology to the rest of the existing rare diseases.

**Other conclusions** that arise from the present study are the following ones. The results obtained from the classification of the potential drugs by the ATC code have been relevant to this research because they have provided a broad idea of the types of drugs that may be the most likely candidates to reposition. The study of the phenotypic similarity has validated the importance of symptom sharing when finding a new use for an existing drug. Moreover, we have uncovered that GDAs are significant elements to be able to verify whether a drug is a potential and effective candidate to be used as a new treatment for a disease different from the original indication of that drug. This implies that the measurement of GDAs can be considered for future studies as an indicator capable of differentiating between better or worse cases of drug repositioning.

However, we have found some **limitations**. The most important one is the lack of research on rare diseases, which directly derives from a lack of associated data. For other types of diseases, the number of disease–drug relationships is superior, which has led us to not being able to validate many of the computationally obtained drugs with the scientific literature. Moreover, rare diseases have a limited number of evidenced associations with biological features, what makes it also difficult to generate these new hypotheses.

In order to tackle these problems, we suggest the following **future lines**. We would like to perform a similar analysis with a larger number of rare diseases. Additionally, applying more complex strategies on the data available on DISNET, such as graph neural networks (GNNs) for the prediction of new relationships focused on repurposing, would be a favourable next step. Along these lines, another fundamental point would be the improvement in the scientific literature validation process. The potential drugs that have been obtained computationally are manually validated with the literature, being a time-consuming and tedious task. Hence, it would be helpful to automatize the clinical trials search. Another future line to be explored, and given the obtained results, probably one of the most enlightening, is the use of biological pathways as a source of repurposing information. We would like to consider biological pathways as a way to find future candidates, since pathways could play a more important role than the one currently played by drug target genes in some cases.

The main endpoint of this research has been to demonstrate the great potential of the developed computational strategies as well as data-driven methods for the search for possible candidate treatments for rare diseases. Furthermore, emphasis has been placed on the existing misinformation on rare diseases which has hindered these computational processes as well as the obtaining of more favourable results.

## Figures and Tables

**Table 1:** Number of genes and symptoms of the 13 rare diseases listed.

CUIs	Disease Name	N. Genes	N. Symptoms
C0011195	Dejerine–Sottas syndrome	31	10
C0023944	Locked-In Syndrome	1	17
C0024054	Lown–Ganong–Levine syndrome	1	6
C0024901	Diffuse cutaneous mastocytosis	1	237
C0027877	Congenital neuronal ceroid lipofuscinosis	38	52
C0036391	Schwartz–Jampel syndrome	23	5
C0265202	Seckel syndrome	15	4
C0268059	Neonatal hemochromatosis	1	43
C0549463	X-Linked Lymphoproliferative Disorder	11	1
C0751337	X-Linked Emery–Dreifuss Muscular Dystrophy	44	32
C0869083	Dahlberg–Borer–Newcomer syndrome	12	2
C1852146	Vibratory urticaria	1	11
C0796280	Acromegaloid facial appearance syndrome	1	90

**Table 2:** The four computational drug-repositioning approaches developed are summarized here. Each case’s number in the table represents the number of drugs involved.

Diseases	Approaches					
	Triples	T. Target	Direct DR	Paths	All	TT.DDR.P *
Dejerine–Sottas syndrome	6	17	17	2	0	2
Locked-In Syndrome	46	0	0	80	44	80
Lown–Ganong–Levine syndrome	0	0	0	965	965	965
Diffuse cutaneous mastocytosis	7	0	0	4	2	4
Congenital neuronal ceroid lipofuscinosis	2	0	0	48	2	48
Schwartz–Jampel syndrome	533	0	30	15	10	14
Seckel syndrome	6	2	2	0	0	2
Neonatal hemochromatosis	2	0	0	91	0	91
X-Linked Lymphoproliferative Disorder	0	0	7	1	1	1
X-Linked Emery–Dreifuss Muscular Dystrophy	10	0	0	126	10	126
Dahlberg–Borer–Newcomer syndrome	1	8	0	0	0	8
Vibratory urticaria	2	0	0	0	2	0
Acromegaloid facial appearance syndrome	35	2	2	0	2	2

* TT = Triples target; DDR = Direct Drug Repositioning; P = Paths.

**Table 3:** Summary of the process to obtain the final number of potential drugs to treat the rare diseases under study. For each disease, the first column shows the number of drugs obtained through the approaches; the second column shows how many of these drugs have been found in the scientific literature; the third column is the number of drugs that can be used to treat the disease; and finally, the last column represents the number of toxic drugs that can produce the disease.

Diseases	Computational Drugs	Drugs Clinical Trials	Drugs Effects	Drugs Toxic
Dejerine–Sottas syndrome	2	2	1	1
Locked-In Syndrome	80	11	2	9
Diffuse cutaneous mastocytosis	4	4	3	1
Congenital neuronal ceroid lipofuscinosis	48	7	5	2
Schwartz–Jampel syndrome	14	1	1	0
Seckel syndrome	2	1	0	1
Neonatal hemochromatosis	91	8	3	5
X-Linked Lymphoproliferative Disorder	1	0	0	0
X-Linked Emery–Dreifuss Muscular Dystrophy	126	15	11	4
Dahlberg–Borer–Newcomer syndrome	8	8	0	8
Vibratory urticaria	2	2	0	2
Acromegaloid facial appearance syndrome	2	1	1	0

**Table 4:** Results of the phenotypic similarity comparison between rare and non-rare diseases with potential drugs versus DISNET disease pairs.

Disease	PS. DISNET Diseases (Mean Jaccard)	PS. Rare—Non-Rare Diseases (Mean Jaccard)	*p*-Value
Dejerine–Sottas syndrome	0.0466	0.0904	0.0112
Schwartz–Jampel syndrome	0.0500	0.8425	0.0000
Seckel syndrome	0.0607	0.0312	0.0173
Dahlberg–Borer–Newcomer syndrome	0.0396	0.1996	0.0000
Acromegaloid facial appearance syndrome	0.0471	0.0319	0.0000

**Table 5:** Results of the disease–gene association in each of the rare diseases studied according to the target gene of the selected potential drug.

Disease	Drug	Gen Symbol	DSI	DPI	GDA
Dejerine–Sottas syndrome	Dexamethasone	NR0B1	0.512	0.621	0.02
	Thalidomide	PTGS2	0.338	0.897	0.02
	Thalidomide	TNF	0.263	0.966	0.02
Schwartz–Jampel syndrome	Carvedilol	VEGFA	0.298	0.897	0.3
Seckel syndrome	Caffeine	ATM	0.401	0.862	0.02
Dahlberg–Borer–Newcomer syndrome	Sorafenib	BRAF	0.352	0.793	0.2
	Fostamatinib				
	Vemurafenib				
	Encorafenib				
	Regorafenib				
	Dabrafenib mesylate				
	Sorafenib tosylate				
	Dabrafenib				
	Fostamatinib	ICK	0.602	0.621	0.2
Acromegaloid facial appearance syndrome	Glyburide	ABCC9	0.59	0.517	0.3

## Data Availability

The data used for this research can be found at the following link: https://medal.ctb.upm.es/internal/gitlab/b.otero/computational_approaches_dr_rare_diseases (accessed on 13 July 2022).
